# Validity and reliability of the international physical activity questionnaire short form in Chilean adults

**DOI:** 10.1371/journal.pone.0291604

**Published:** 2023-10-03

**Authors:** Teresa Balboa-Castillo, Sergio Muñoz, Pamela Serón, Omar Andrade-Mayorga, Pamela Lavados-Romo, Nicolás Aguilar-Farias

**Affiliations:** 1 Department of Public Health, Faculty of Medicine, Universidad de La Frontera, Temuco, Chile; 2 Cardiometabolic and Nutritional Epidemiology Research Center (EPICYN), Faculty of Medicine, Universidad de La Frontera, Temuco, Chile; 3 Department of Rehabilitation Sciences & CIGES, Faculty of Medicine, Universidad de La Frontera, Temuco, Chile; 4 Department of Preclinical Sciences, Faculty of Medicine, Universidad de La Frontera, Temuco, Chile; 5 Department of Physical Education, Sports and Recreation, Universidad de La Frontera, Temuco, Chile; Universitatea de Medicina si Farmacie Victor Babes din Timisoara, ROMANIA

## Abstract

**Purpose:**

This study aimed to determine the test-retest reliability and concurrent validity of the International Physical Activity Questionnaire Short Form (IPAQ-SF) in Chilean adults.

**Methods:**

A cross-sectional validation study was carried out on 161 adults aged between 35 and 65, selected from a population-based study in Temuco, Chile. IPAQ-SF was completed twice, seven days apart, to analyze the test-retest reliability with the intraclass correlation coefficient (ICC). Objective PA was assessed by accelerometry (ActiGraph GT3X+) for seven consecutive days. Intraclass correlation coefficients were used to determine the reliability. Spearman correlation coefficients (rho) and Bland-Altman plots were calculated to assess validity.

**Results:**

144 subjects (52.5 ± 8.8 years, 53.9% men) answered the IPAQ-SF on both occasions and had valid accelerometry data. The IPAQ-SF showed moderate reliability for sitting time (ICC = 0.62), while it was poor for walking (ICC = 0.40), moderate PA (ICC = 0.41), vigorous PA (ICC = 0.48), and total PA (ICC = 0.33). There were weak correlations between IPAQ-SF and accelerometry for sedentary behavior (rho = 0.28, p = 0.0005), walking (rho = 0.11, p = 0.17), moderate PA (rho = 0.13, p = 0.128), vigorous PA (rho = 0.18, p = 0.03), and total PA (rho = 0.26, p = 0.002).

**Conclusions:**

The results suggest that the IPAQ-SF test and retest would provide an acceptable measure of total SB and MVPA, and a weak correlation between IPAQ-SF and accelerometer.

## Introduction

Strong scientific evidence shows that physical activity (PA) is one of the major factors in the prevention, control, and treatment of chronic non-communicable diseases, like cardiovascular diseases (CVD), diabetes, and some specific cancers [1–4]. For this reason, PA assessment is one of the most important fields of epidemiology and public health. However, PA assessment is currently a challenge in population studies because the more precise methods (double-labeled water, indirect calorimetry, and motion sensors) are complex and expensive. Questionnaires are the most common instruments used to assess PA in public health because they are cost-effective and easy to administer in large sample sizes [5]. Several questionnaires are available, and one of the most applied is the International Physical Activity Questionnaire (IPAQ) [6]. The IPAQ short form (IPAQ-SF) is the most used version and has been used worldwide to evaluate PA indirectly throughout the last seven days in different activity intensities. Also, has been validated across the world in different languages and it was found to have fair to moderate agreement with accelerometer-measured physical activity [7–9]. Its reliability has been tested in various countries and populations, finding good to excellent reliability [6]. Moreover, the IPAQ was developed for global physical activity surveillance to permit comparability among countries, and has shown significant variations according to the socio-cultural context. For this reason, the authors have suggested examining population differences [10–17]. In the Latin American population some studies have explored the validity of IPAQ, but only in the long form [16–18], however the psychometric properties of IPAQ have not been measured in Chile, limiting interpretation and global comparisons.

Thus, this study aimed to determine the test-retest reliability and concurrent validity of the International Physical Activity Questionnaire Short Form (IPAQ-SF) in Chilean adults.

## Methods

### Study design and participants

Participants of the present cross-sectional validation study were selected from a population-based study (CESCAS), a prospective cohort with a multistage probabilistic sampling of participants aged 35–74 years from four mid-sized cities representing the Southern Cone of Latin America were included. A detailed description of the study population and design has been presented elsewhere [19]. The cross-sectional validation study was restricted to a probabilistic sample of 161 adults aged 35–65 from Temuco city, Chile. Data assessment took place between May 2015 and January 2016. Subjects with known Parkinson’s or cerebrovascular diseases were excluded from this study because the accelerometer sensor may register measurement errors. The study was approved by the scientific ethics committee at the Universidad de La Frontera, Temuco, Chile (Project Approval N° 053/2014), and all participants provided written informed consent before the study.

### Physical activity assessment by IPAQ short form (IPAQ-SF)

Self-reported PA data were collected using the Spanish (USA) version of IPAQ-SF (available at https://sites.google.com/view/ipaq/download). IPAQ-SF includes seven questions compared with the 27 of the IPAQ-long version.

The IPAQ was administered in face-to-face interviews in two separate visits, seven days between one interview (IPAQ1) and another (IPAQ2). The IPAQ-SF asks participants to report PA performed for at least 10 minutes during the last seven days. Respondents were asked to report time spent in PA three intensities: walking, moderate, and vigorous in all daily life domains. Examples of activities that represent each intensity were provided. We use the Compendium of physical activities to specify activities by MET intensity [20].

The IPAQ also asks daily sitting time to indicate sedentary behavior (SB). Total weekly PA was estimated using IPAQ scoring protocol. The total time for each intensity and activity type was calculated. Total energy expenditure per week (MET min week) was derived from the time spent in each activity intensity multiplied by its estimated metabolic equivalent of task (MET). The IPAQ scoring protocol assigns the following MET values for walking, moderate-intensity, and vigorous-intensity activities: 3.3 METs, 4.0 METs, and 8.0 METs, respectively [21].

### Physical activity assessment by accelerometry

Objective PA was assessed by accelerometry using ActiGraph GT3X+ (Actigraph, Pensacola, FL, USA), an electronic device validated and widely used in biomedical research [22]. All participants were instructed to use the accelerometer with oral and written information by a research assistant. The subjects were asked to wear the accelerometer on the right hip, attached to an elastic belt, during waking hours for seven consecutive days. They were instructed to remove the device only for sleep and water-based activities. After seven consecutive days, the participants returned the accelerometer device and answered the IPAQ2. The ActiLife 6 (version 6.13.1, ActiGraph, Pensacola, FL, USA) data analysis software was used to initialize the accelerometers and download data. An automated algorithm was used to identify wearing and non-wearing periods (i.e., 90 minutes of consecutive inactivity) [23]. A day was considered valid if the participant wore the device for at least 10 hours. Accelerometer data were included in the analyses if participants had valid information on at least three weekdays and one weekend day [24]. Each minute (i.e., 60-second epoch) of accelerometer data was classified as sedentary behavior (defined as all activity at <100 activity counts/min), light-intensity (100–1951 counts/min), moderate-intensity (1952–5724 counts/min), or vigorous-intensity activities (5725–9498 counts/min), based on Freedson cut-points for the vector magnitude [25].

### Other variables

We obtained information on sociodemographic and anthropometric variables. Specifically, study participants reported their sex, age, marital status, and educational level. Marital status was categorized into two categories: being married and being unmarried, which also included being single, separated, divorced, and widowed. Educational level was classified into primary, secondary, and university studies. Weight and height were measured under standardized conditions [26]. Body mass index (BMI) was calculated as weight in kilograms divided by square height in centimeters. Waist circumference (WC) was measured using standardized techniques with participants in light clothing using a flexible, inelastic belt-type tape [26].

### Statistical methods

Characteristics of the study population were described in the total sample and by sex. The sociodemographic characteristics of subjects were described with frequency distribution, while anthropometric features were described using means and standard deviations (SD). As physical activity data were not normally distributed, we used nonparametric tests, and data are shown as medians and interquartile ranges. Intraclass correlation coefficients (ICC) were used to assess the test-retest reliability of the IPAQ between one interview (IPAQ1) and another (IPAQ2). The ICC is a recommended estimator to evaluate the magnitude of the relationship between multiple assessments of the same variable [27]. Spearman correlation coefficients (rho) with 95% confidence intervals (CI; derived using Fisher’s z transformation) and Bland-Altman plots with 95% limits of agreement were calculated to assess validity by comparing values between (and within) the IPAQ-SF and accelerometry. Spearman’s rho coefficients were used because physical activity data were not normally distributed. The correlation coefficients were classified as follows: > 0.8, very strong; 0.61–0.80, moderate; 0.30–0.60, fair; and < = 0.30, poor [28]. For all analyses, the significance level was set to p < 0.05. The analyses were performed with STATA (version 14, Stata Statistical Software: College Station, TX: Stata Corp LP) [29].

## Results

The characteristics of the study population are shown in Table 1. Of the 161 volunteers, only 144 participants answered the IPAQ-SF on both occasions (7 days apart) and had valid accelerometry data. The mean age of study participants was 52.5±8.8 years, and 53% were men. Most had secondary or higher education (83%), were overweight (45%), and had obesity (43%).

**Table 1:** Sociodemographic and anthropometric characteristics of the study population.

	Total (N = 144)	Men (N = 77)	Women (N = 67)
**Age**, (years), mean ± SD	52.5 ± 8.8	53.9 ± 7.3	51.0 ± 10.0
**Marital status**, n (%)			
Single/separated/divorced/widow	39 (27.1)	17 (22.1)	22 (33.8)
Married/Informal married	105 (72.9)	60 (77.9)	45 (67.2)
**Educational level**, n (%)			
Primary	24 (16.7)	12 (15.6)	12 (17.9)
Secondary	74 (51.4)	39 (50.7)	35 (52.2)
University	46 (31.9)	26 (33.8)	20 (29.9)
**Anthropometry**, mean ± SD			
Body mass index (kg/m^2^)	29.5 ± 4.2	29.3 ± 3.4	29.7 ± 4.9
Waist circumference (cm)	94.9 ± 10.2	98 ± 8.4	91.4 ± 10.9
**Nutritional status** (BMI, Kg/m2), n (%)			
Normal	17 (11.8)	8(10.4)	9(13.4)
Overweight	65 (45.1)	35 (45.5)	30 (44.8)
Obese	62 (43.1)	34 (44.1)	28 (41.8)

The descriptive data for the IPAQ-SF and accelerometric PA assessment are shown in Table 2. Regarding sedentary behavior for the total sample, on average, participants underestimated sedentary time as measured with the IPAQ-SF compared with accelerometry (3.0 h/day *vs*. 9.3 h/day). Moreover, physical activity was significantly lower when measured with the IPAQ-SF compared with the accelerometry for the total sample, and it was similar in both sexes.

**Table 2:** Data for physical activity measured by IPAQ-SF and accelerometry (ActiGraph GT3X+).

	Total (N = 144)	Men (N = 77)	Women (N = 67)	p Value
**Physical activity**				
**IPAQ-SF**				
Time spent sitting (hours/day)	3.0 (1.0–8.0)	3.0 (1.0–8.0)	4.0 (1.0–8.0)	0.273
Walking (minutes/day)	132.9 (9.3–540)	128.6 (4.3–480)	137.1 (14.3–540)	0.874
Moderate PA (minutes/day)	17.1 (0–248.6)	28.6 (0–308.6)	17.1 (0–205.7)	0.055
Vigorous PA (minutes/day)	0 (0–43.9)	0 (0–102.8)	0 (0–25.7)	0.071
**ACCELEROMETRY**				
Sedentary behavior (hours/day)	9.3 (6.7–12.1)	9.4 (6.5–13.3)	8.9 (6.9–11.3)	0.249
Light PA (minutes/day)	192.3 (117.9–270.5)	193.1 (117.8–269.0)	189.9 (132.1–284.8)	0.869
Moderate PA (minutes/day)	99.3 (61.5–160.4)	98.6 (61.4–185.2)	100.4 (60.4–140.8)	0.869
Vigorous PA (minutes/day)	10.9 (5.5–35.5)	13.4 (5.3–52.4)	10.1 (5.6–23.7)	0.014
Steps per day	14735 (0–23031)	15597 (0–24158)	13530 (0–22193)	0.001
MVPA (minutes/day)	112.8 (72.9–181.3)	115.6 (70.4–210.0)	109.0 (74.4–160.5)	0.249

Data are presented as median and percentiles (P5-P95). PA: physical activity, MVPA: moderate to vigorous physical activity.

Finally, accelerometry detected differences between men and women in vigorous PA (13.4 [5.3–52.4] vs. 10.1 [5.6–23.7] min/day; p = 0.014) and steps per day (15597 [0–24158] vs. 13530 [0–22193] steps/day; p = 0.001), which IPAQ did not (Table 2).

### Reliability

The test-retest reliability between IPAQ1 and IPAQ2 and their different domains determined by the intraclass correlation coefficient (ICC) fluctuated between 0.40 and 0.62 (Table 3). Moderate reliability was found for sitting time (ICC = 0.62), while it was poor for walking (ICC = 0.40), moderate PA (ICC = 0.41), vigorous PA (ICC = 0.48), and total PA (ICC = 0.33). Male participants showed higher ICC than women for sitting time and moderate PA, while women had higher ICC for vigorous PA than men (Table 3).

**Table 3:** Test-retest reliability of the IPAQ-SF.

IPAQ	Total		Man		Women	
	ICC	CI 95%	ICC	CI 95%	ICC	CI 95%
**Sitting time** (hours/day)	0.62	(0.35–0.88)	0.73	(0.48–0.97)	0.42	(0.09–0.75)
**Walking** (minutes/day)	0.40	(0.18–0.61)	0.38	(0.09–0.66)	0.39	(0.09–0.69)
**Moderate PA** (minutes/day)	0.41	(0.17–0.65)	0.51	(0.24–0.79)	0.33	(0.01–0.66)
**Vigorous PA** (minutes/day)	0.48	(0.10–0.85)	0.33	(0.00–0.75)	0.49	(0.01–0.98)
**Total PA** (MET·min·wk^-1^)	0.33	(0.00–0.73)	0.19	(0.00–1.00)	0.21	(0.00–0.96)

ICC: intraclass correlation coefficients; CI: confidence interval

### Validity

The validity of the IPAQ-SF was tested by Spearman correlation coefficients (Rho) for PA intensities assessed by IPAQ-SF and accelerometry (Table 4). There were weak correlations between IPAQ-SF and accelerometer measures for sedentary behavior (Spearman r = 0.28, p<0.005), walking (Spearman r = 0.11, p = 0.17), moderate PA (Spearman r = 0.13, p = 0.13), vigorous PA (Spearman r = 0.18, p = 0.03), and total PA (Spearman r = 0.26, p = 0.002) (Table 4).

**Table 4:** Spearman correlation coefficients (Rho) for physical activity intensities assessed by IPAQ-SF and accelerometer by sex.

IPAQ-SF	Accelerometer	Total		Man		Women	
		rho	P-value	rho	P-value	rho	P-value
Sitting time (minutes/day)	Sedentary behavior (minutes/day)	0.28	0.0005	0.21	0.0670	0.38	0.001
Time walking (minutes/day)	Time in light and moderate PA (minutes/day)	0.11	0.1700	0.20	0.0700	0.03	0.79
Time walking (minutes/day)	Time in moderate PA (minutes/day)	0.24	0.0030	0.42	0.0001	0.02	0.85
Time walking and moderate (minutes/day)	Time in moderate PA (minutes/day)	0.30	0.0004	0.47	<0.0001	0.05	0.67
Time in moderate PA (minutes/day)	Time in moderate PA (minutes/day)	0.13	0.1300	0.14	0.2400	0.13	0.29
Time in vigorous PA (minutes/day)	Time in vigorous PA (minutes/day)	0.18	0.0300	0.13	0.2400	0.13	0.29
Time in total PA METs 3.0+ (minutes/day)	Time in total MVPA (minutes/day)	0.26	0.0020	0.42	0.0001	0.03	0.78

PA: physical activity, MVPA: moderate to vigorous physical activity. Rho: Spearman rank correlation coefficient.

**Fig 1:**
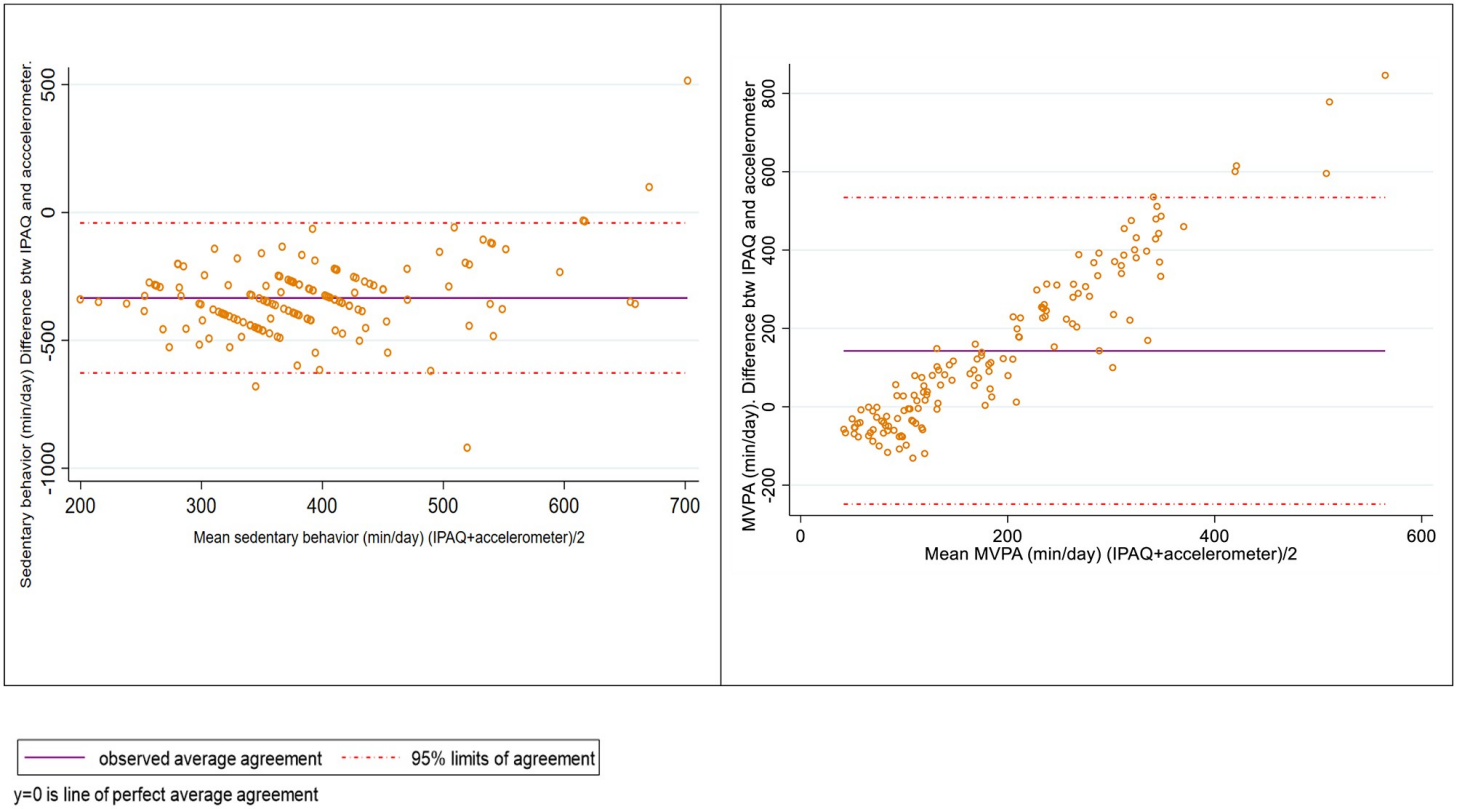
Bland–Altman plots for the agreement of data assessed with IPAQ and with accelerometers.

Bland-Altman plots for the agreement of data assessed by IPAQ-SF and accelerometry for sedentary behavior (SB) and total moderate to vigorous PA (MVPA) are shown in Fig 1.

The IPAQ-SF underestimates time compared with accelerometers for measurement of time spent performing SB. Most values of the difference between SB measurements are negative and grouped in a band, indicating a constant distance between the self-report and the accelerometer measurement (Fig 1 **panel A**). The data suggest that adjusting the self-report might decrease the measurement bias.

Regarding MVPA in IPAQ-SF, this is underestimated in subjects with a low level of PA but overestimated in subjects with a high level of PA. Moreover, a non-zero slope was observed, which suggests PA level-dependent differences between the self-report and accelerometry (Fig 1 **panel B**). Therefore, the higher the PA, the greater the difference between the measurements in the self-report.

## Discussion

The results of the current study suggest that IPAQ-SF test and retest reliability would provide an acceptable measure of total SB and MVPA in Chilean adults. Concerning validity between IPAQ-SF and accelerometry, our results revealed a weak correlation for SB and MVPA. Additionally, it was found that IPAQ-SF underestimates MVPA in subjects with a low level of PA and overestimates MVPA in adults with high levels of PA. The IPAQ-SF systematically underestimates the sitting time for measurement of time spent performing SB.

Our results are consistent with other studies made in different populations and countries. Evidence shows that PA questionnaires have moderate reproducibility and low validity compared with objective methods [6]. Lee *et al*. performed a systematic review of twenty-three validation studies, which reported a weak correlation between total physical activity level measures by IPAQ-SF and objective measurement in most studies. This systematic review reported a correlation from 0.09 to 0.39 [14]. Moreover, Medina *et al*. found modest reliability and poor validity in a convenience sample of Mexican adults [18]. Likewise, the Hungarian cohort of the European Physical Activity and Sport Monitoring System project found excellent test-retest reliability but low concurrent validity [8]. In addition, Tran *et al*., reported acceptable validity for IPAQ-SF in Asian adults, and they found that the instrument can classify responders as achieving or not achieving the recommended PA levels [30]. A review and meta-analysis that included 20 studies carried out in the European Union found that test-retest reliability was moderately high, and validity was moderate in most used international PA questionnaires (PAQs), including the IPAQ-SF [31]. Considering physical activity is multidimensional and complex [32], no simple measures adequately reflect the self-reported physical activity. Also, in extensive epidemiological studies, IPAQ- SF can predict the quality of life and mortality [33].

Related to sedentary behavior, in concordance with a systematic review carried out in adults in the European Union, our results revealed a reliable measure and weak validity for the single-item sedentary behavior question of IPAQ-SF [9]. However, these results are expected because evidence shows that single-item questions significantly underreport sitting time compared to multi-item questionnaires [34].

In order to recommend an instrument to measure physical activity and sedentary behavior in the population, evidence suggests that the intraclass correlation coefficient (ICC) or the weighted Kappa must be greater than or equal to 0.5 compared with objective methods for physical activity measurement [35]. Our study found acceptable correlations for repeated administration of IPAQ-SF in sedentary time and vigorous PA (greater than or equal to 0.5) and a low correlation between questionnaire and accelerometry in Chilean adults.

Finally, a review carried out by Helmerhorst *et al*., [36] that compared properties to the other questionnaires shows similar results to IPAQ- SF, demonstrating the difficulty of measuring physical activity based on self-reported questionnaires, which still needs to be more precise to report on the level of physical activity without bias.

The main strength of the study is the methodology used to allow comparability with similar studies, following the same protocol as other validity IPAQ- SF studies [7, 10, 11, 13, 14, 37]. Also, to our knowledge, this is the first study to validate the IPAQ-SF in the Chilean adult population. The volunteers were recruited from the community using multistage sampling, allowing to capture of participants with different sociodemographic characteristics with a wide range of movement features and behaviors. Although a device-derived measure (in some cases referred to as an objective measure) was used as a reference for the validation, it should be considered that accelerometers may miss information from upper body movement, weight loading, and surface slope. Accelerometers also may not capture changes in body posture or detect the difference between standing or sitting, which could also affect the difference between PA measures by questionnaire and accelerometry in free-living environments. Also, to reduce measurement error, we applied the IPAQ-SF twice, one week apart, because it is long enough for the participants to forget their previous responses but not long enough to avoid changes in physical activity behavior. Finally, we meet recommendations to improve PA measurement derived from self report [38].

## Conclusions

Based on these results, the IPAQ-SF test and retest would provide an acceptable measure of total SB and MVPA but weak correlation between IPAQ-SF and accelerometry. In this sense, the IPAQ-SF did not provide acceptable levels of validity compared with the gold-standard measure (accelerometry) and did not meet the minimum acceptable standard of 0.5 for objective activity measuring devices. However, the reliability of the IPAQ-SF assessed by test and retest was acceptable and the strength of the relationship according to correlation coefficients was fair to moderate. These results imply that the relationship between multiple assessments of physical activity by IPAQ-SF was reasonable, and the questionnaire could be a valuable instrument to measure changes in physical activity when the objective is to compare this behavior with a baseline measurement assessed by IPAQ-SF. A significant result is that the IPAQ performs differently when this is applied to sedentary and active subjects. Regarding MVPA, the IPAQ-SF, underestimated physical activity in subjects with a low level of PA but overestimated it in subjects with a high level of PA.
